# Assessment of 16S rRNA sequencing for analysis of circulating microbial DNA in colorectal cancer patients–proof of concept and early changes during experimental chemoimmunotherapy

**DOI:** 10.3389/fmicb.2026.1802448

**Published:** 2026-06-08

**Authors:** Adriana M. Sanabria, Tonje Bjørnetrø, Anniken J. Fuglestad, Sebastian Meltzer, Anne Helene Køstner, Torben Lüders, Christian Kersten, Anne Hansen Ree

**Affiliations:** 1 Department of Oncology, Akershus University Hospital, Lørenskog, Norway; 2 Institute of Clinical Medicine, University of Oslo, Oslo, Norway; 3 Center for Cancer Treatment, Sørlandet Hospital, Kristiansand, Norway; 4 Department of Clinical Molecular Biology, Akershus University Hospital, Lørenskog, Norway

**Keywords:** 16S rRNA sequencing, circulating microbial DNA, colorectal cancer, immunothearpy, plasma, serum

## Abstract

**Background:**

Circulating microbial DNA (cmDNA) is an emerging biomarker in cancer, yet its analytical and clinical utility remains to be validated.

**Methods:**

This study establishes a proof-of-concept for cmDNA sequencing and analysis using 16S rRNA amplicons in colorectal cancer (CRC) patient samples. Two cohorts were analyzed – the Test cohort (*n* = 11) comparing plasma and serum from early-stage colon cancer patients to determine the optimal sample type, and the METIMMOX cohort (*n* = 11) of patients with newly diagnosed metastatic CRC to explore the initial dynamics of cmDNA alterations during experimental therapy. The METIMMOX trial investigated alternating oxaliplatin-based chemotherapy and nivolumab in metastatic microsatellite-stable CRC, the major CRC entity essentially considered unresponsive to immune checkpoint inhibitors. For the metastatic CRC patients, plasma was used for cmDNA analysis at baseline and following the initial chemoimmunotherapy.

**Results:**

Both plasma and serum were suitable for cmDNA profiling; however, plasma was preferred due to higher numbers of bacterial reads and lower proportion of unassigned reads. Compared to METIMMOX patients with long-lasting treatment response, exhibiting a stable initial cmDNA composition, the patients unresponsive to chemoimmunotherapy showed an increase in alpha diversity (observed amplicon sequence variants: *p* = 0.014, Chao1 species richness indices: *p* = 0.010, Shannon community evenness indices: *p* = 0.004) over the short period of time until treatment failure. Likewise, beta diversity estimation (by Bray-Curtis dissimilarity indices) indicated that the early treatment course influenced the cmDNA composition differently in patients with and without response.

**Conclusion:**

This study demonstrates the feasibility of cmDNA sequencing in CRC patients and highlights its potential to uncover treatment-related microbial shifts that may serve as non-invasive biomarkers of therapeutic response or resistance.

## 1 Introduction

A notable feature of colorectal cancer (CRC) is its reliance on the gut microbiota, affecting not only the primary tumor and its microenvironment but also the metastatic colonization of distant organs (Cass and White, 2023; Mignini et al., 2024). Alterations in microbial composition, known as dysbiosis, can provoke detrimental responses in colonic epithelial cells, metastatic target organs, and the host immune system, promoting tumorigenesis, CRC progression, and impaired responses to chemotherapy and immunotherapy (You et al., 2022; Zhao et al., 2023).

Gut dysbiosis can lead to increased intestinal permeability, facilitating bacterial translocation and the presence of circulating microbial DNA (cmDNA) in the bloodstream (Tripathi et al., 2018; Pietrzak et al., 2023). In cancer patients, immune suppression may permit these microbes to persist in the circulation, potentially contributing to chronic inflammation, immune modulation, and tumor progression (You et al., 2022; Zhou et al., 2023; Bautista et al., 2026). Recent cmDNA discoveries have highlighted that Firmicutes, a dominant phylum of the gut microbiome, may distinguish between individuals with resectable CRC and cancer-free persons (Vicente-Valor et al., 2025), suggesting its potential utility as a diagnostic tool. In addition, *Fusobacterium nucleatum* may distinguish between early-stage and metastatic CRC (Koliarakis et al., 2024). Together, these conditions provide a rationale for investigating cmDNA dynamics as a non-invasive biomarker in CRC patients.

However, cmDNA analysis faces considerable technical hurdles, largely due to its low abundance and susceptibility to contamination. Detected mainly as short DNA fragments, cmDNA can arise either from degraded microbial cells released into circulation or from actively secreted nucleic acids produced by viable microbes (Kowarsky et al., 2017; Castillo et al., 2019). The concentration of cell-free bacterial DNA fragments in whole blood, plasma, and serum is generally very low, which complicates detection and analysis. Moreover, the field lacks consensus on the most suitable specimen type for cmDNA profiling (You et al., 2022).

Colorectal cancer-associated cmDNA signatures hold potential diagnostic and prognostic value; however, their dynamics, especially concerning treatment efficacy, are not fully understood (You et al., 2022). Increasing evidence suggests that the composition and diversity of the gut microbiota can influence both the response and tolerability of immune checkpoint inhibitors (ICIs) (Wang et al., 2018; Hayase and Jenq, 2021; Thompson et al., 2022; Wardill et al., 2022; Natarelli et al., 2024). Moreover, the diversity of CRC-associated cmDNA has been identified as a predictive marker for response to chemotherapy combined with adoptive T-cell immunotherapy (Yang et al., 2021).

The present study has two main objectives. First, we evaluated paired serum and plasma samples from early-stage colon cancer patients to determine which biofluid, if any, provides superior performance for cmDNA analysis using 16S rRNA amplicon sequencing. Identifying the optimal sample type may improve the accuracy and reliability of cmDNA data in CRC. Next, we explored cmDNA dynamics in patients with newly diagnosed metastatic CRC during their initial experimental treatment with chemoimmunotherapy. Characterizing these dynamics may offer valuable insights relevant to personalized treatment strategies.

## 2 Materials and methods

### 2.1 Study participants and procedures

The required approvals were given by the Regional Committee for Medical and Health Research Ethics of South-East Norway (2019/28744 and 2017/1850), the Norwegian Medicines Agency (17/12752), and the institutional review boards. The studies were conducted under the Declaration of Helsinki. All patients provided written informed consent. The Test cohort consisted of paired serum and plasma samples collected before curative surgery of 11 patients with early-stage, non-metastatic (stage I–III) colon cancer. The METIMMOX cohort (registered with ClinicalTrials.gov, NCT03388190; by 2 January 2018) consisted of plasma samples from 11 patients with metastatic (stage IV) microsatellite-stable CRC undergoing experimental first-line treatment consisting of alternating 2 cycles each of oxaliplatin-based chemotherapy and the ICI nivolumab, as detailed previously (Ree et al., 2024). Plasma samples were collected immediately before treatment initiation (defined as the Pre variable) and after completion of the initial 2 cycles of chemotherapy and 2 cycles of nivolumab (∼2 months of treatment; the Post variable). Patients were classified as Responders (median progression-free survival 32.9 months, range 20.7–41.6) or Non-Responders (median progression-free survival 2.1 months, range 1.9–2.6). Patient and disease characteristics for the METIMMOX cohort are listed in Supplementary Table 1. For validation purposes, plasma samples from 3 METIMMOX patients who, at the 2-months sampling time point, had received chemotherapy only were analyzed.

### 2.2 Patient samples

**FIGURE 1:**
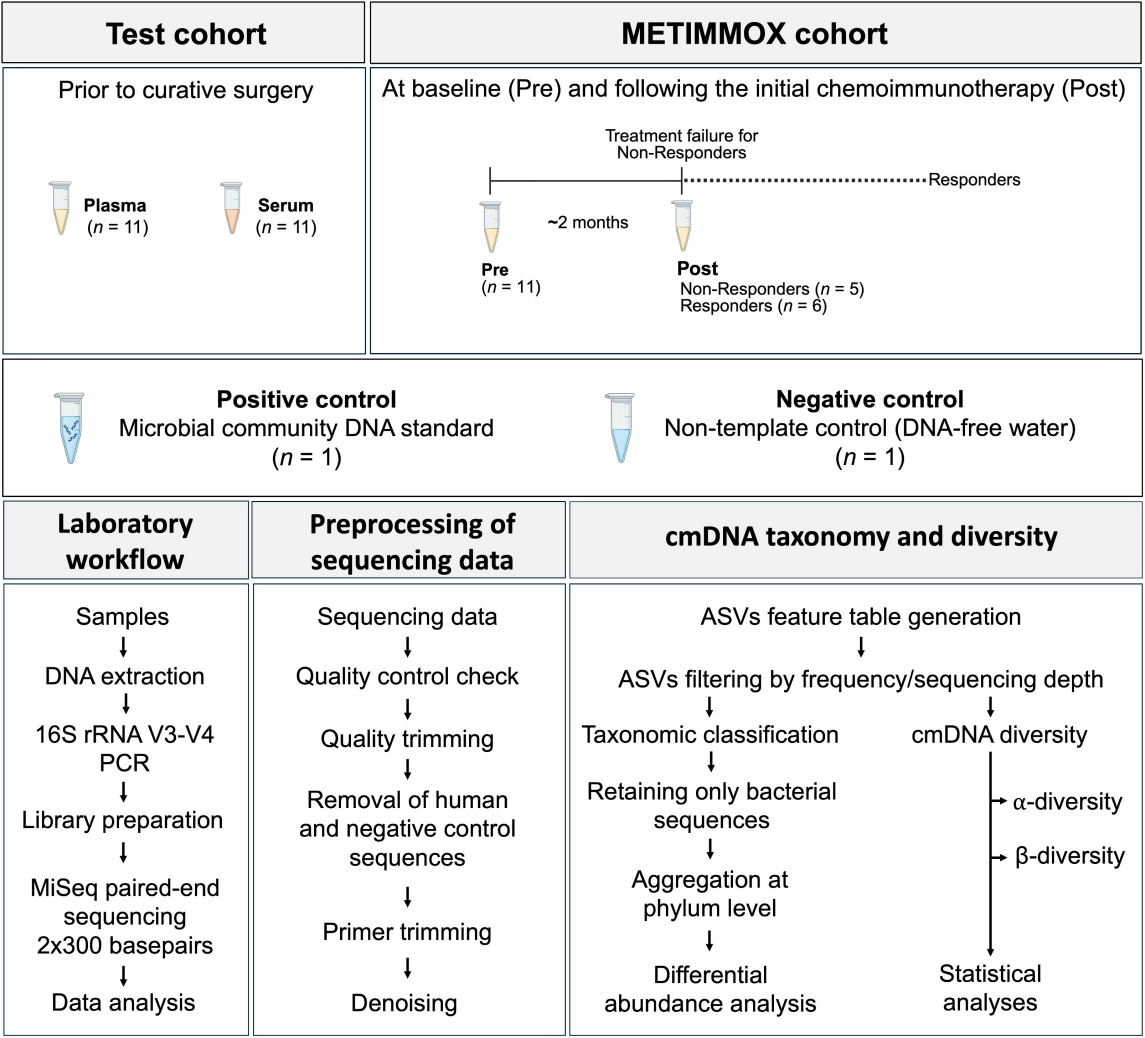
Circulating microbial DNA (cmDNA) in colorectal cancer patients – the study design. Overview of patient and sample collections in the Test cohort and METIMMOX cohort, the laboratory workflow, the preprocessing of sequencing data, and cmDNA taxonomy and diversity analyses of the amplicon sequence variants (ASVs).

Plasma was prepared in EDTA collection tubes by centrifugation at 2,000 *g* for 10 min. Serum collection tubes were gently inverted five times and left at room temperature for 45 min before centrifugation under the same conditions as plasma. All samples were aliquoted and stored at −80 °C until further analysis. DNA was extracted from 400 μL of plasma or serum using the QIAamp UCP Pathogen Mini Kit (Qiagen) and 25 μL DNA-free water (Molzym) as elution buffer. Total DNA concentration was measured using the Qubit dsDNA HS Assay Kit (Thermo Fisher Scientific), and the quality was assessed using Nanodrop ND 1000 spectrophotometer (Thermo Fisher Scientific). A commercially available microbial community DNA standard (ZymoBIOMICS™) was included as a positive control and a non-template (water) control was used during DNA amplification to monitor potential contamination. Additionally, we attempted broad-range 16S rRNA qPCR for absolute bacterial DNA quantification; however, amplification signals were consistently close to the assay’s limit of detection and therefore not suitable for reliable quantification in our samples. Figure 1 provides an overview of the laboratory and *in silico* methods for the downstream 16S rRNA amplicon sequencing for cmDNA analysis.

### 2.3 16 S rRNA gene amplification and sequencing

The V3-V4 region of the microbial 16S rRNA gene was amplified using the primers 341-F (5′-ACTCCTACGGGAGGCAGCAG-3′) and 806-R (5′-GGACTACHVGGGTWTCTAAT-3′), synthesized with 5′ Illumina overhang adapter sequences (Klindworth et al., 2013). The PCR was performed in 25-μL reactions containing 11 μL of DNA, 12.5 μL of KAPA HiFi HotStart ReadyMix (KAPA Biosystems), and 0.75 μL of each forward and reverse oligonucleotide primer (10 μM) (Klindworth et al., 2013). The PCR cycling conditions included an initial denaturation at 95°C for 3 min, followed by 35 cycles at 95°C for 30 s, 55°C for 30 s, and 72°C for 30 s, with a final elongation step at 72°C for 5 min. The amplified PCR products were subjected to library preparation following the MiSeq system guide (Illumina) with modifications at the indexing PCR step, including adjustments of the DNA volume (10 μL) and number of PCR cycles (15 cycles). Dual indices and adapter sequences were incorporated using the Nextera XT Index Kit (Illumina). Library size distribution and quality were confirmed on a Bioanalyzer DNA 1000 chip (Agilent Technologies). The library was loaded at 0.5 pM with 20% (v/v) Illumina PhiX control, and sequencing was conducted on the Illumina MiSeq platform using the MiSeq Reagent Kit v3 with 2 × 300-basepair read lengths.

### 2.4 Quality control and preprocessing of sequencing data

The sequencing data were demultiplexed and subjected to quality control in FastQC v0.11.9^1^ and Trimmomatic v0.30.2 (Bolger et al., 2014). The negative controls consisting of nuclease-free water included during PCR amplification were sequenced alongside biological samples. Contaminant DNA was filtered out using Bowtie aligner integrated in FastQ Screen v0.13.0 (Wingett and Andrews, 2018). To remove human sequences, the reads were mapped to the human genome GRCh38.p13 (GCF_000001405.39). Reads detected in the negative control were excluded from the dataset to minimize potential contamination. Sequences were further processed in the microbiome analysis package QIIME2 v2022.2 (Bolyen et al., 2019). Primer sequences were removed using Cutadapt (Martin, 2011) and denoising was performed with the DADA2 algorithm (Callahan et al., 2016). Following preprocessing, the remaining DNA sequences were too short to cover the full 16S rRNA V3-V4 region; hence, downstream analyses were conducted using forward reads only. Finally, each sequence was truncated at position 150 to generate the amplicon sequence variants (ASVs) feature table. Features with a read count below 10 and those present in a single sample only were excluded to reduce noise of the downstream analyses. As only a limited number of sequenced negative controls were available, prevalence-based contaminant identification methods could not be applied. Therefore, we adopted a conservative filtering strategy combining negative-control-based filtering and taxonomic curation to reduce the influence of potential contaminants.

### 2.5 Taxonomic classification

The ASV taxonomy assignment was carried out via the QIIME2 feature-classifier classify-sklearn using a pre-trained classifier for the V3-V4 region^2^ based on the SILVA reference database (119 SSU Ref NR 99 341F/805R release) (Quast et al., 2013). To remove possible contaminants, sequencing artifacts, and non-target groups, ASVs were filtered to retain only bacterial sequences with at least phylum-level classification, excluding unclassified and non-bacterial sequences. Taxonomic profiles were analyzed at the phylum level using the Phyloseq package in R. Cyanobacteria were excluded to minimize the influence of likely environmental or reagent contaminants. Feature counts were aggregated to the phylum level^3^ and relative abundances were calculated per sample.

### 2.6 Estimation of bacterial community diversities

Samples were normalized to the minimum sequencing depth of 3,990 reads for the Test cohort and 1,170 reads for the METIMMOX cohort. Alpha diversity (within-sample cmDNA diversity) was assessed by the number of observed ASVs, the Chao1 species richness index, and the Shannon community evenness index. Beta diversity (across-samples differences in cmDNA composition) was evaluated by the Bray-Curtis dissimilarity index. The diversity analyses were done in Phyloseq v1.44.0 (McMurdie and Holmes, 2013).

### 2.7 Statistical analysis

Categorical variables were presented as percentages and continuous variables as mean with standard deviation (SD) or median with interquartile range (IQR), as appropriate. Data distribution was assessed using Shapiro-Wilk test. Within-group (paired) comparisons were done by paired samples *t*-test for normally distributed data or Wilcoxon matched-pairs signed-rank test for non-normally distributed data. Between-group (independent) comparisons were done by independent samples *t*-test for normally distributed data or Mann-Whitney U test for non-normally distributed data, as appropriate.

Interactions between the METIMMOX sampling time (the Pre or Post variables) and treatment outcome (the Responders or Non-Responders variables) were assessed using two-way repeated-measures ANOVA. Likewise, interactions between any antibiotic exposure, treatment outcome, and changes in alpha diversity (Post-Pre) were assessed in two-way ANOVA models including interaction terms, after verification of model assumptions. The findings were corroborated by *post hoc* pairwise comparisons adjusted using Tukey’s method. The normality of data and residuals was evaluated using the Shapiro-Wilk test. Serum levels of C-reactive protein (CRP), assessed at every study visit in the METIMMOX trial, were log-transformed and analyzed using linear mixed-effects models with sampling time and treatment outcome as fixed effects and patient identity as a random effect. Associations between CRP and alpha diversity were assessed using similar models.

Differential abundance analysis was initially performed in DESeq2 v1.40.2 (Love et al., 2014), which offers high sensitivity and is well suited for longitudinal designs. Results were re-evaluated with the more conservative ALDE × 2 framework (Fernandes et al., 2014), which is designed to reduce false positive findings. For the latter, multiple testing correction was performed using the Benjamini-Hochberg false discovery rate (FDR). As ALDE × 2 produced broadly similar patterns with lower sensitivity, DESeq2 was retained as the primary method and ALDE × 2 was used to confirm the robustness of the findings. Principal Coordinates Analysis (PCoA) was performed in Phyloseq v1.44.0 to visualize patterns of similarity and dissimilarity among samples. Beta diversity was tested using single and paired Permutational Multivariate Analysis of Variance (PERMANOVA) with the adonis2 function in Vegan v2.6.4^4^. All analyses applied a significance level of *p* < 0.05. Data analyses and visualizations were conducted in R v4.3.1^5^ and GraphPad Prism9 v9.5.1 (GraphPad Software Inc.).

## 3 Results

### 3.1 Sequencing performance

Using the described sequencing protocol on the Test cohort, the average cluster density of 846,000/mm^2^ yielded approximately 9,750 megabases and 15.8 million reads with a Q30 score exceeding 70%, indicating high-quality raw data. Of these, plasma samples contributed 6.1 million reads and serum samples 5.1 million reads; the remaining reads were unassigned or belonged to the control samples. The sequencing depth was more uniform for the plasma samples, yielding a median read number of 541,635 (IQR 369,676–703,757), while the serum samples had a median read number of 304,218 (IQR 156,447–1,084,235). As depicted in Supplementary Figure 1, following preprocessing of the raw data, a similar proportion of reads was retained (plasma mean 21.2%, SD 6.4 *versus* serum mean 19.9%, SD 8.3; *p* = 0.69, paired *t*-test), a finding maintained on taxonomic classification at the domain level (plasma mean 16.5%, SD 5.4 *versus* serum mean 14.7%, SD 6.2; *p* = 0.44, paired *t*-test) and after excluding unclassified and non-bacterial sequences (plasma mean 6.9%, SD 3.8 *versus* serum mean 4.0%, SD 2.6; *p* = 0.075, paired *t*-test). Ultimately, mean 1.1% (SD 1.1) of the plasma reads and mean 1.6% (SD 1.1) of the serum reads were retained for bacterial diversity analyses.

The sequencing of the METIMMOX plasma samples provided an average cluster density of 871,000/mm^2^, produced 9,944 megabases and 16.1 million raw reads, and had a Q30 score above 68%. The median read number was 241,020 (IQR 73,391–617,987). Despite using the forward reads only, the positive controls run in the sequencing of both the Test cohort and METIMMOX cohort aligned with the expected microbial composition (Supplementary Table 2). The negative controls of the two runs indicated low background interference with common contaminants (Supplementary Table 3). Taken together, cmDNA was detectable in both plasma and serum, supporting the use of both biofluids for the analysis of this low-yield analyte.

### 3.2 Microbial composition of the paired plasma and serum samples

**FIGURE 2:**
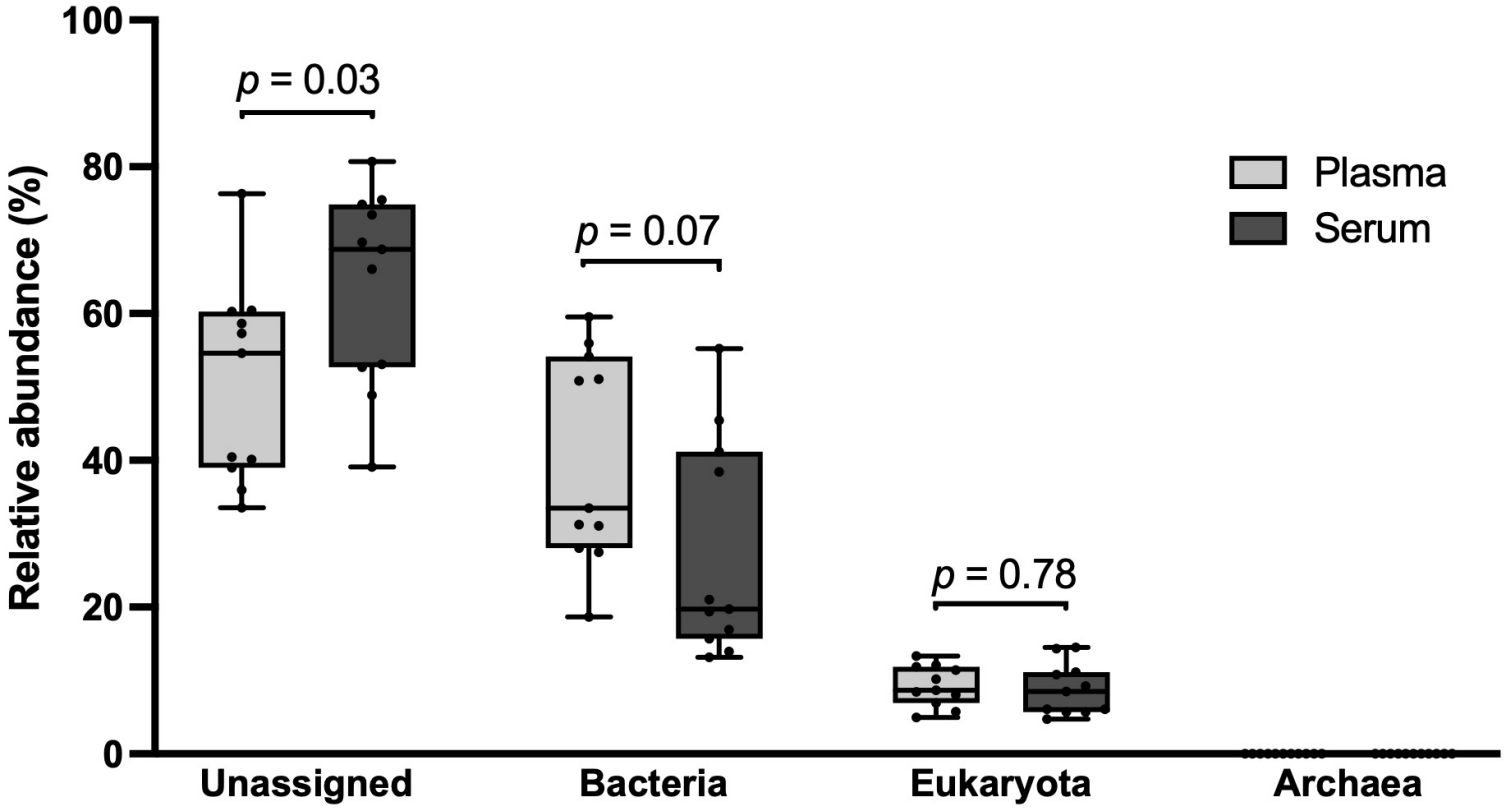
Taxonomic classification at the domain level – comparison of plasma and serum; the Test cohort. The proportion of reads classified as Bacteria, Eukaryota, or Archaea, and those remaining unassigned. Boxes represent medians and interquartile ranges; *p*-values were obtained using the Wilcoxon test.

As illustrated in Figure 2, taxonomic classification at the domain level revealed a higher proportion of unassigned reads in serum (median 68.8%, IQR 52.7–74.9) compared with plasma (median 54.6%, IQR 39.0–60.3; *p* = 0.042, Wilcoxon test). Plasma samples showed a higher proportion of bacterial reads (median 33.5%, IQR 28.0–54.2) than serum (median 19.8%, IQR 15.7–41.2; *p* = 0.067, Wilcoxon test), consistent with the greater absolute bacterial read counts observed in plasma (*p* = 0.014, Wilcoxon test). The proportion of eukaryotic reads was similar (plasma median 8.7% *versus* serum median 8.5%; *p* = 0.78, Wilcoxon test), while archaeal reads were essentially absent in both.

After removing Cyanobacteria, present in trace amounts in plasma (0.9%) and serum (1.5%), the two biofluids were comparable in taxonomic cmDNA composition at the phylum level (Supplementary Figure 2; group-level mean relative abundances are shown). The composition was dominated by Actinobacteriota (plasma 32.7%, serum 31.6%), Firmicutes (plasma 21.4%, serum 28.1%), Proteobacteria (plasma 24.9%, serum 19.3%), and Deinococcota (plasma 19.3%, serum 19.6%). Bacteroidota (plasma 1.8%, serum 1.5%) were less abundant. Paired comparisons revealed no significant differences between plasma and serum for any phylum (*p* > 0.05). Differential abundance analysis by DESeq2 likewise showed no significant compositional differences of phyla between plasma and serum (adjusted *p*-values > 0.05; Supplementary Figure 3), a finding corroborated by ALDE × 2 (FDR > 0.05).

**FIGURE 3:**
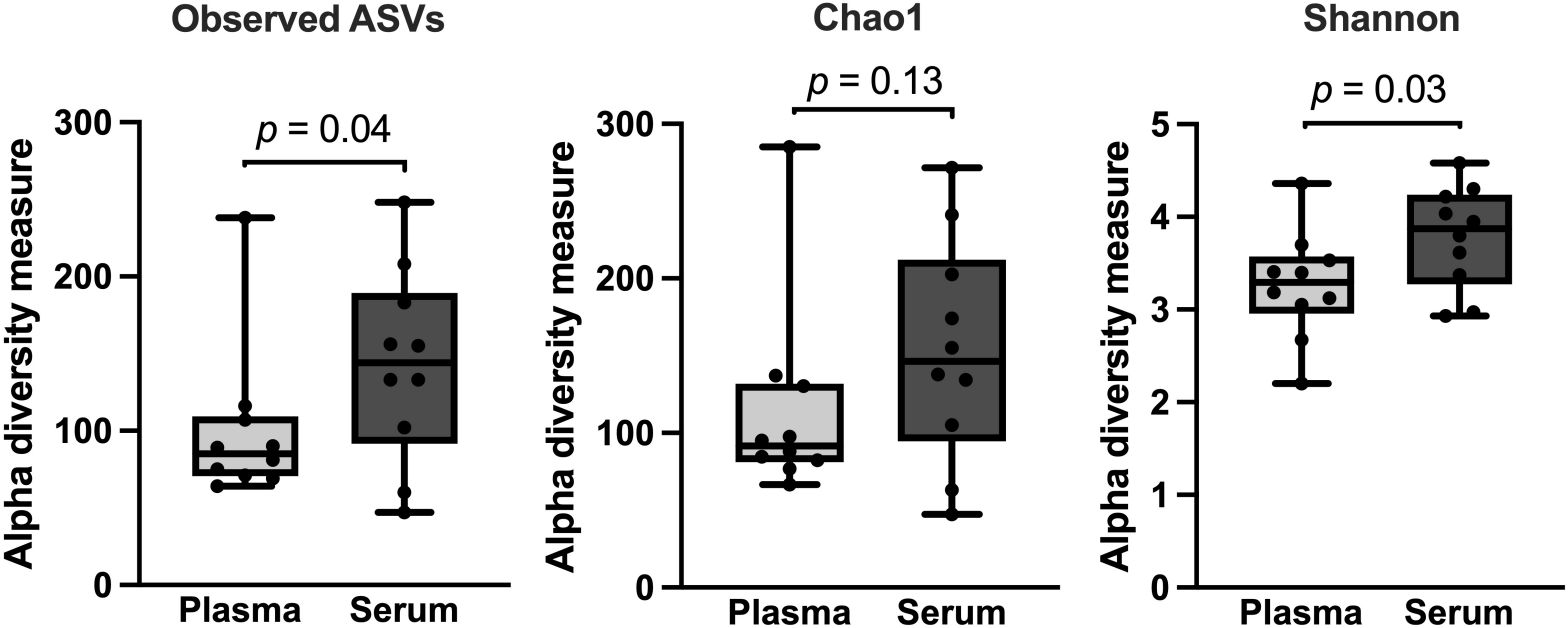
Alpha diversity indices of the circulating microbial DNA – comparison of plasma and serum; the Test cohort. The number of observed amplicon sequence variants (ASVs), the Chao1 species richness, and the Shannon community evenness. Boxes represent medians and interquartile ranges; *p*-values were obtained using paired samples *t*-test or Wilcoxon test, as appropriate.

Following the data filtering for bacterial ASVs (while preserving ASV-level resolution) and normalization to the minimum sequencing depth of 3,990 reads, 736 ASVs were identified across 21 plasma and serum samples (one serum sample was excluded due to an insufficient number of reads). For the alpha diversity metrics (Figure 3), serum had higher number of observed ASVs (median 144.0, IQR 109.8–176.3 *versus* plasma median 85.0, IQR 72.0–102.8; *p* = 0.037, Wilcoxon test) and higher Shannon community evenness index (mean 3.8, SD 0.55 *versus* plasma mean 3.3, SD 0.58; *p* = 0.029, paired *t*-test), while the Chao1 species richness indices were comparable (serum median 146.4, IQR 112.3–195.4 *versus* plasma median 91.6, IQR 81.0–132.0; *p* = 0.13, Wilcoxon test).

The beta diversity was moderate to high (Bray-Curtis dissimilarity indices of 0.58–0.92) across the serum and plasma samples. By PCoA, no distinct clusters were found for sample type or patient identity (Supplementary Figure 4). Likewise, PERMANOVA did not attribute beta diversity to sample type (R^2^ = 5.3%; *p* = 0.44) or to patient identity (R^2^ = 5.3%; *p* = 0.35). However, the latter explained a larger proportion of the overall cmDNA diversity (R^2^ = 49.4%), although this did not reach statistical significance (*p* = 0.15).

In summary, plasma yielded higher numbers of bacterial reads but serum exhibited higher alpha diversity measures. Still, the two biofluids performed comparably for most of the variables analyzed, and the choice of sample type was not critical for the fidelity of cmDNA data.

### 3.3 Treatment-related cmDNA alterations

For the metastatic CRC patients, plasma was used for cmDNA analysis at baseline (designated Pre) and following the initial experimental chemoimmunotherapy (designated Post). The cohort comprised Responders (*n* = 6) and Non-Responders (*n* = 5); classification and metrics for progression-free survival are reported in Methods.

**FIGURE 4:**
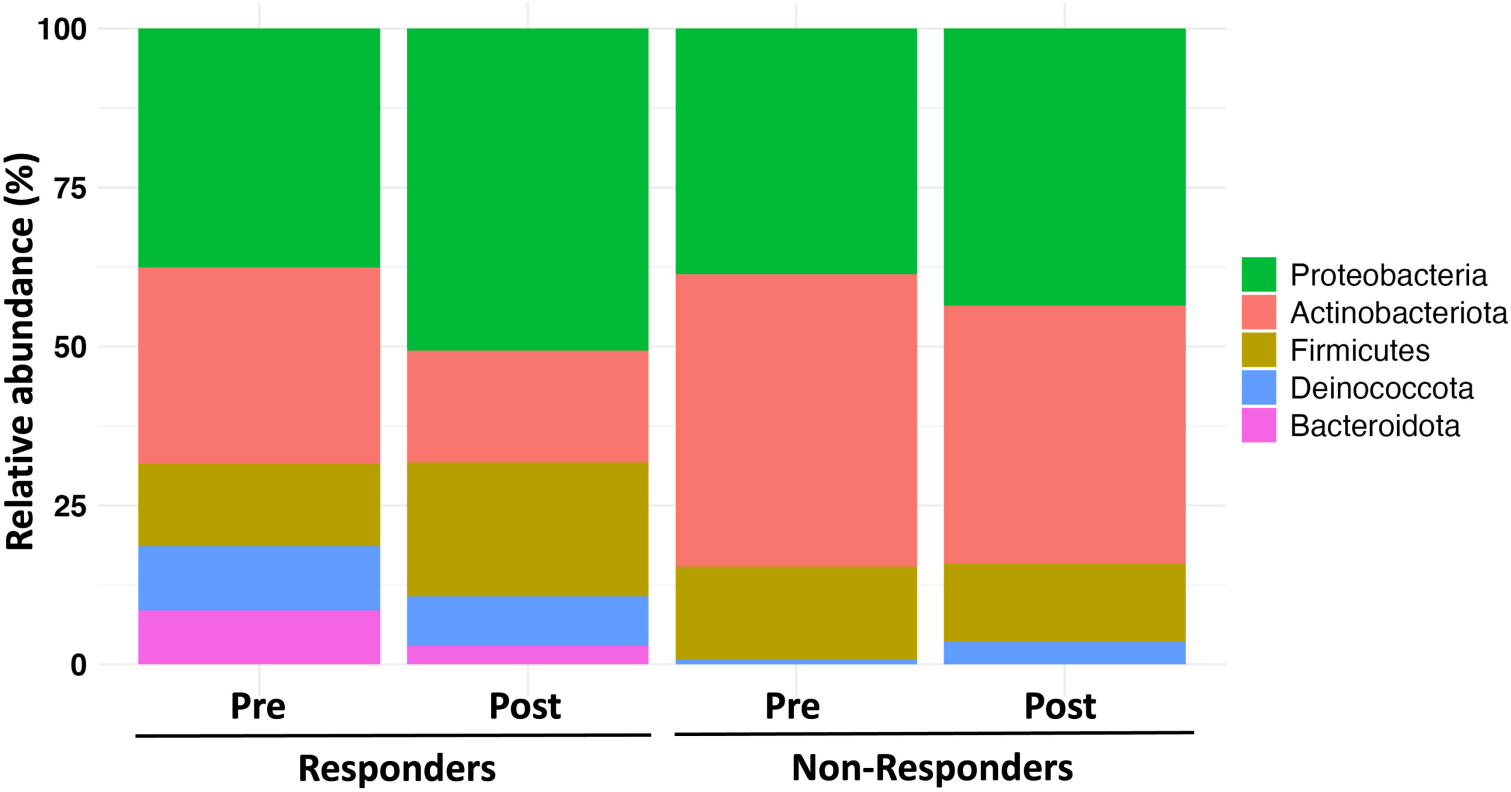
Relative abundance of the major plasma bacterial phyla at baseline (Pre) and following the initial chemoimmunotherapy (Post) for Responders and Non-Responders; the METIMMOX cohort. The dominant phyla of the circulating microbial DNA; group-level mean relative abundances.

Differential abundance analysis by DESeq2 of all Pre and Post samples together (Supplementary Figure 5) showed that in Responders (adjusted *p*-values for the comparisons *versus* Non-Responders), Bacteroidota (log_2_ fold-change = 7.58, *p* = 0.034) and Proteobacteria (log_2_ fold-change = 1.02, *p* = 0.034) were more abundant. In contrast, Actinobacteriota, Deinococcota, and Firmicutes were comparable (*p* > 0.1) in patients with and without treatment response. With the more conservative ALDE × 2, however, none of the phyla showed significant differences between Responders and Non-Responders (FDR > 0.05). The relative abundance at baseline (Pre) and after the initial experimental therapy (∼2 months of treatment; Post) are depicted in Figure 4 (group-level mean relative abundances are shown), demonstrating that the same phyla dominated at baseline for both Responders and Non-Responders. These were the same phyla that also dominated in serum and plasma of the Test cohort, validating the analytical approach for CRC patients. Furthermore, within both Responders and Non-Responders, the overall composition of phyla, as assessed by DESeq2, remained largely unaltered during the initial treatment course (adjusted *p*-values > 0.05; Supplementary Table 4), a finding corroborated by ALDE × 2 (FDR > 0.05). In plasma from 3 METIMMOX patients who, at the 2-months sampling time point had received chemotherapy only, a similar taxonomic composition was retrieved – Proteobacteria (46.8%), Firmicutes (43.1%), and Actinobacteriota (9.2%), while Deinococcota (0.4%) and Bacteroidota (0.5%) were almost undetectable.

**FIGURE 5:**
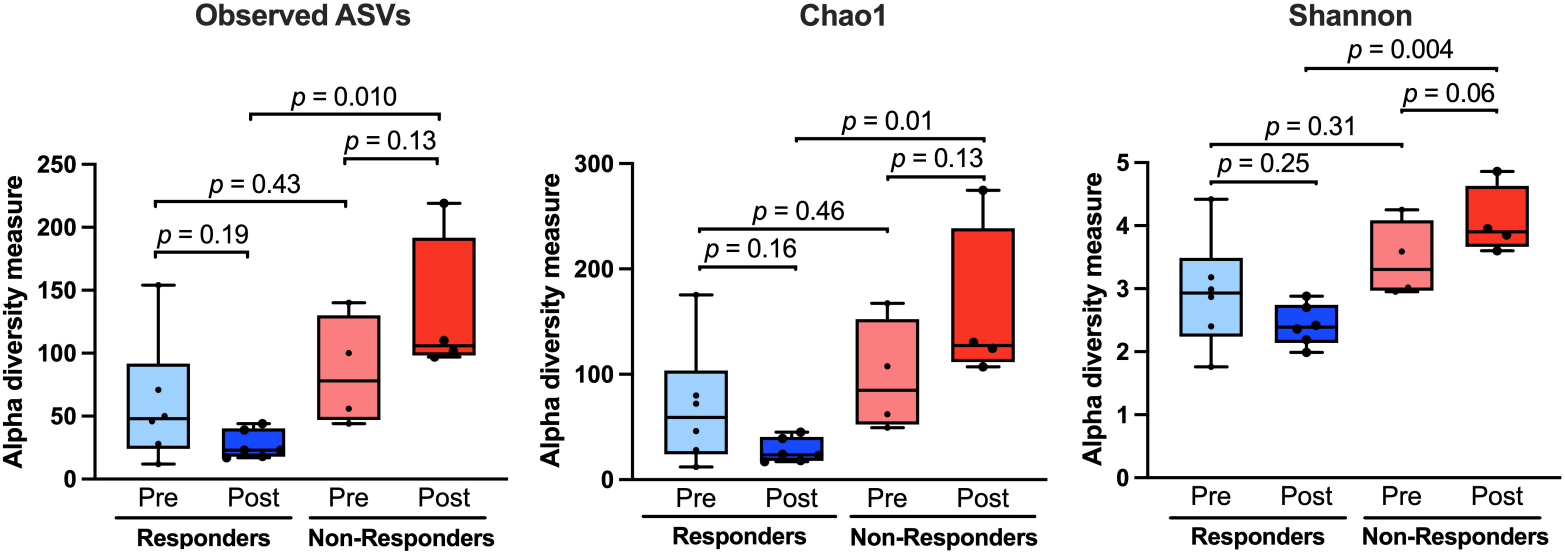
Alpha diversity indices of the circulating microbial DNA – comparison of Responders and Non-Responders at baseline (Pre) and following the initial chemoimmunotherapy (Post); the METIMMOX cohort. The number of observed amplicon sequence variants (ASVs), the Chao1 species richness, and the Shannon community evenness. Boxes represent medians and interquartile ranges; *p*-values were obtained using paired samples *t*-test or Wilcoxon test (comparisons at Pre or Post) or independent samples *t*-test or Mann-Whitney U test (comparisons of Pre versus Post), as appropriate.

As shown in Figure 5, Responders and Non-Responders to the experimental chemoimmunotherapy exhibited comparable alpha diversity measures at Pre. At Post, however, Non-Responders featured significantly higher plasma diversity than Responders, as reflected by the number of observed ASVs (median 106.0, IQR 100.8–137.3 *versus* median 23.0, IQR 19.3–35.0; *p* = 0.014, Mann-Whitney U test), the Chao1 species richness index (median 127.4, IQR 120.2–166.4 *versus* median 23.5, IQR 19.3–35.3; *p* = 0.010, Mann-Whitney U test), and the Shannon evenness index (mean 3.8, SD 0.63 *versus* mean 2.7, SD 0.69; *p* = 0.0040, independent *t*-test).

Interaction analysis (two-way repeated-measures ANOVA) confirmed that Pre-to-Post diversity increased in Non-Responders while decreasing in Responders with regard to observed ASVs (*p* = 0.022) and the Chao1 index (*p* = 0.011) but not the Shannon index (*p* = 0.077), supporting a differential cmDNA responsiveness in the two patient groups. *Post hoc* pairwise comparisons verified that all alpha diversity metrics were higher in Non-Responders than Responders at Post (observed ASVs, *p* = 0.0016; Chao1, *p* = 0.0014; Shannon, *p* = 0.0020) with no other pairwise differences, again supporting a differential cmDNA diversity response during the initial treatment course in the two patient groups.

Tumor-induced systemic inflammation is a fundamental feature of poor-prognosis early-stage and advanced CRC (Park et al., 2016; Sjoquist et al., 2018). During the initial chemoimmunotherapy, CRP levels were higher in Non-Responders than in Responders (*p* = 0.032), and higher CRP was associated with both increased observed ASVs (*p* = 0.037) and a higher Shannon index (*p* = 0.029), but not with the Chao1 index (*p* = 0.068). There were no statistically significant associations between patients’ antibiotic exposure during the initial study treatment and changes in alpha diversity indices.

**FIGURE 6:**
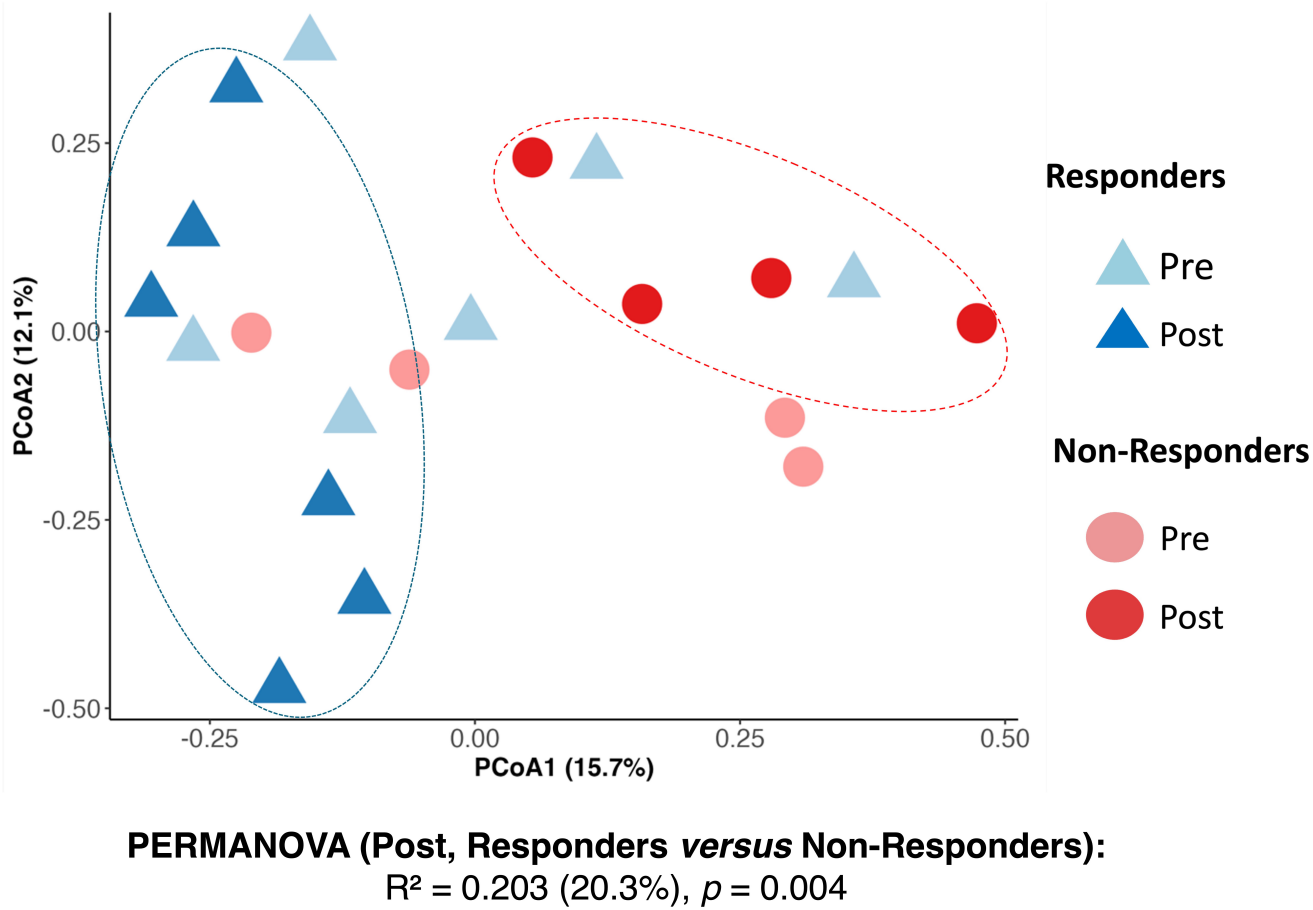
Beta diversity of the circulating microbial DNA in Responders and Non-Responders at baseline (Pre) and following the initial chemoimmunotherapy (Post); the METIMMOX cohort. Principal Coordinates Analysis (PCoA) score plot based on the Bray-Curtis dissimilarities. Each point represents the composition of the plasma bacterial DNA of one patient at one of the two sampling times. The numbers in brackets are the PCoA scores of the total variation. Ellipses indicate 95% confidence intervals for the respective Post samples. PERMANOVA values are indicated.

Beta diversity estimation (by Bray-Curtis dissimilarity indices) revealed discrete cmDNA community compositions in Responders and Non-Responders at Post, with a significant variance attributed to the treatment outcome variable (R^2^ = 20.3%, *p* = 0.0040; by PERMANOVA), again indicating that the early treatment course influenced the cmDNA composition differently in the two patient groups. Consistently, PCoA based on Bray-Curtis distances indicated separation of Responders and Non-Responders (Figure 6).

In summary, patients with metastatic CRC unresponsive to an experimental chemoimmunotherapy regimen showed an increase in cmDNA diversity over the short period leading to treatment failure. In contrast, patients with long-lasting treatment response maintained a stable initial cmDNA composition, including unaltered relative abundances of the dominant bacterial phyla.

## 4 Discussion

In this study, we provide proof of concept for cmDNA analysis in CRC patients using 16S rRNA sequencing. First, by comparing plasma and serum, we established an optimized workflow that accounts for key pre-analytical and analytical variables to preserve signal fidelity. Next, we explored potential cmDNA alterations in patients with metastatic CRC receiving experimental chemoimmunotherapy and observed an early increase in cmDNA diversity in patients with treatment failure but not among patients with long-lasting treatment response. Given that the metastatic CRC cohort consisted of only 11 subjects, these results should be interpreted as strictly exploratory and hypothesis-generating rather than definitive.

The clinical utility of cmDNA depends on overcoming technical challenges, foremost among them contamination. Methodological rigor is essential in low-biomass microbiome research because the validity of observations depends on distinguishing true biological signal from background noise and reagent or environmental contaminants. The analytical difficulty is compounded by the fragmented nature of cmDNA (fragments are often less than 150 basepairs long) and the fact that microbial DNA represents only a minute fraction of total cell-free DNA in the circulation (Kowarsky et al., 2017; Poore et al., 2020). In cancer patients, microbial reads have been reported to constitute as little as 0.009%–0.012% of total sequencing reads (Chen et al., 2024). In our study, less than 2% of plasma and serum reads were retained after stringent data filtering. Moreover, cmDNA is prone to degradation during sample handling, which further complicates detection and taxonomic assignment.

Additionally, data validity has been re-emphasized following the discovery of major errors in a prominent report (Poore et al., 2020). Independent re-examinations identified two principal flaws that undermined the original conclusions – contamination of bacterial genome databases with human sequences, which caused extensive misclassification of human reads as bacterial, and data normalization artifacts that introduced spurious cancer-specific signals absent from the raw data (Gihawi et al., 2023). These appraisals underscore the need for rigorous contamination control and a transparent analytical workflow to ensure reproducible and valid cmDNA results (Santacroce et al., 2024).

In light of these challenges, we implemented several complementary measures to maximize data reliability, with particular focus on reducing the influence of contaminants. The first was bioinformatic decontamination, applying a robust computational pipeline to identify and remove contaminant sequences. This approach parallels models developed to distinguish tissue-resident microbiota from contaminants in large-scale datasets and has been essential for deriving reliable tissue-specific microbial profiles (Dohlman et al., 2021). The second was sequencing depth normalization, where all samples were normalized to a minimum sequencing depth to standardize information content and reduce variability due to technical causes. The third was the approach of analyzing paired samples collected at baseline and following the initial experimental chemoimmunotherapy from the same individuals, reducing the influence of interindividual variation and enabling more confident attribution of observed alterations to the therapeutic intervention. By maintaining high sequencing quality metrics and applying comprehensive controls and filtering procedures, we ensured that the resulting cmDNA profiles likely represented *bona fide* biological signals.

A key methodological step was selecting the optimal sample matrix for cmDNA analysis. Pre-analytical variables such as the type of matrix have been shown to influence the quantity and quality of recoverable cmDNA (Bronkhorst et al., 2019; Cheng et al., 2019). Our comparative evaluation favored plasma over serum because plasma yielded greater read retention, fewer unassigned taxa, and higher inter-sample consistency. This choice aligns with prior evidence identifying plasma as the preferred matrix for cmDNA and related liquid biopsy analyses (Arneth, 2018; Castillo et al., 2019; Chen et al., 2024). However, serum samples demonstrated higher alpha diversity indices, suggesting that serum-based analysis may capture complementary aspects of circulating microbial complexity to those observed in plasma.

A notable finding was the change in cmDNA diversity in patients with treatment failure to the experimental chemoimmunotherapy (Non-Responders), but not in those with long-lasting treatment response (Responders). Alpha diversity metrics increased significantly in Non-Responders, which is somewhat unexpected, as higher microbial diversity is typically associated with healthier gut ecosystems (Chen et al., 2021; Messaritakis et al., 2024), and high baseline cmDNA diversity has been linked to response to adoptive T-cell immunotherapy in metastatic CRC (Yang et al., 2021). Beta diversity analysis nevertheless corroborated that the early treatment course influenced cmDNA composition specifically in Non-Responders. Although alpha and beta diversity measures are highly susceptible to technical noise in low-biomass datasets, our findings raise the hypothesis that a more diverse microbial pool in the circulation reflects greater chemotherapy-induced disruption of the mucosal gut barrier in Non-Responders, facilitating microbial translocation into the bloodstream (Zhou et al., 2025). Gut dysbiosis has been implicated in promoting barrier breakdown, chronic inflammation, and immune evasion (Bautista et al., 2026), all of which could plausibly influence cmDNA diversity, but these mechanisms remain speculative in the context of our data. Of note, Non-Responders exhibited higher CRP levels than Responders, and elevated CRP was associated with increased alpha diversity, observations consistent with tumor-induced systemic inflammation, an adverse feature of CRC, although causality cannot be inferred. Finally, nivolumab (the ICI component of the METIMMOX regimen) could theoretically modulate immune surveillance and clearance of translocated microbial DNA in Responders, thereby limiting systemic microbial diversification. The discrepancy between our findings and previous reports highlights the complexity and context dependence of microbial biomarkers in cancer immunotherapy.

Beyond the measures of diversity, the compositional analysis suggested distinct microbial signatures associated with treatment response. Responders appeared to be enriched for Bacteroidota and Proteobacteria compared to Non-Responders, although these trends did not reach significance when using the more conservative ALDE × 2 statistical algorithm. Notably, certain taxon-level differences detected by DESeq2 were not reproduced by ALDE × 2, underscoring that the compositional shifts observed in our dataset should be regarded as tentative signals. Taxa within Bacteroidota and Proteobacteria include organisms with immunostimulatory capacities that may promote antitumor immunity (Zhao et al., 2023; Khan et al., 2025). Moreover, patients with locally advanced rectal cancer obtaining major pathological response to neoadjuvant chemoradiotherapy have been reported to exhibit significantly lower bacterial burden and a stronger adaptive immune profile in the baseline tumor than patients without such response (Ajami et al., 2025; Morgan et al., 2025).

A strength of the study is the demonstration that 16S-based cmDNA profiling can be technically implemented in plasma samples from CRC patients, including longitudinal sampling in metastatic disease. The use of paired samples (Pre and Post) from patients with metastatic disease enabled assessment of dynamic microbial shifts in relation to therapeutic response, a feature rarely addressed in cmDNA research. Methodologically, the study implemented comprehensive contamination control measures along with stringent bioinformatic filtering procedures to ensure the reliability of low-biomass microbial signals. However, residual background contamination cannot be completely excluded and remains an inherent limitation of cmDNA studies. Moreover, the sample size was small, reflecting the exploratory and resource-intensive nature of cmDNA sequencing and limiting statistical power and generalizability. Because insufficient overlap between forward and reverse reads necessitated retention of only high-quality forward reads, sequencing depth was reduced and the 16S rRNA amplicon approach constrained taxonomic resolution to the phylum level, precluding further species-level identification and direct functional inference.

In conclusion, our study provides evidence that cmDNA is a technically feasible and biologically informative analyte, with potential utility as a non-invasive biomarker for treatment monitoring and for assessing systemic host-microbe interactions in CRC patients. However, this hypothesis needs to be challenged and confirmed in larger sample cohorts.

## Data Availability

The datasets presented in this article are not readily available because they contain human-derived sequencing data that are stored securely at Akershus University Hospital and are subject to legal and ethical restrictions under the General Data Protection Regulation (GDPR) of the European Union, due to the potential risk of re-identification. Requests to access the datasets should be directed to the corresponding author (adriana.m.moreno@ahus.no) and are subject to approval by the Data Privacy Officer and, where applicable, the Regional Committee for Medical and Health Research Ethics of South-East Norway.

## Footnotes

1
https://www.bioinformatics.babraham.ac.uk/projects/fastqc/

2
https://github.com/anweshmaile/silva-138_classifiers

3
https://www.rdocumentation.org/packages/phyloseq/versions/1.16.2/topics/tax_glom

4
https://cran.r-project.org/web/packages/vegan/vegan.pdf

5
https://www.stat.auckland.ac.nz/~ihaka/downloads/R-paper.pdf
