# Supplementary material for: Assessment of 16S rRNA sequencing for analysis of circulating microbial DNA in colorectal cancer patients–proof of concept and early changes during experimental chemoimmunotherapy
Source: Front Microbiol. 2026 Jun 8;17:1802448. doi: 10.3389/fmicb.2026.1802448 (PMC13283987; doi:10.3389/fmicb.2026.1802448)
Supplement: Supplementary file 1 [file Supplementary_file_1.docx]

Supplementary Material

# Supplementary Figures


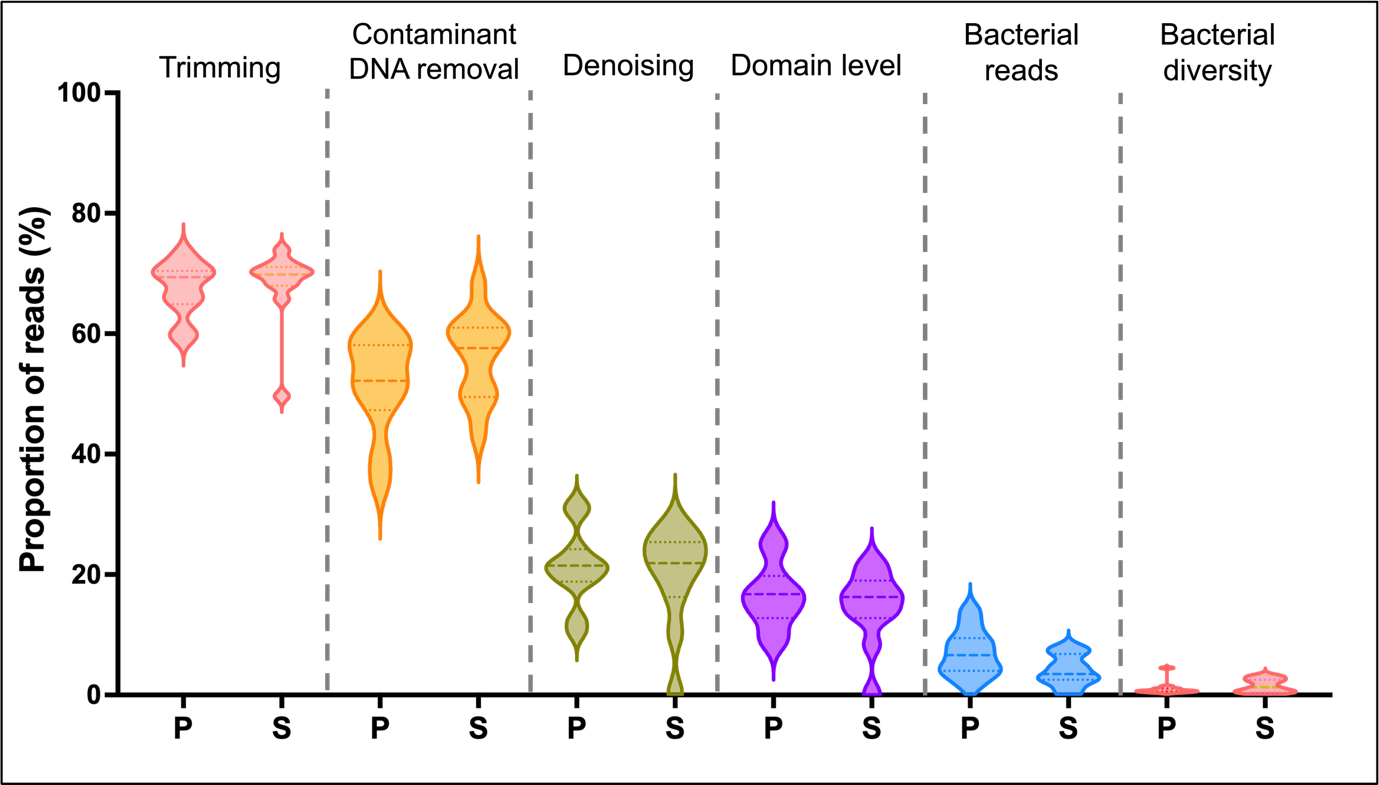


**Supplementary Figure S1. Proportions of read retention from plasma (P) and serum (S) at different analytical phases in the workflow; the Test cohort.**


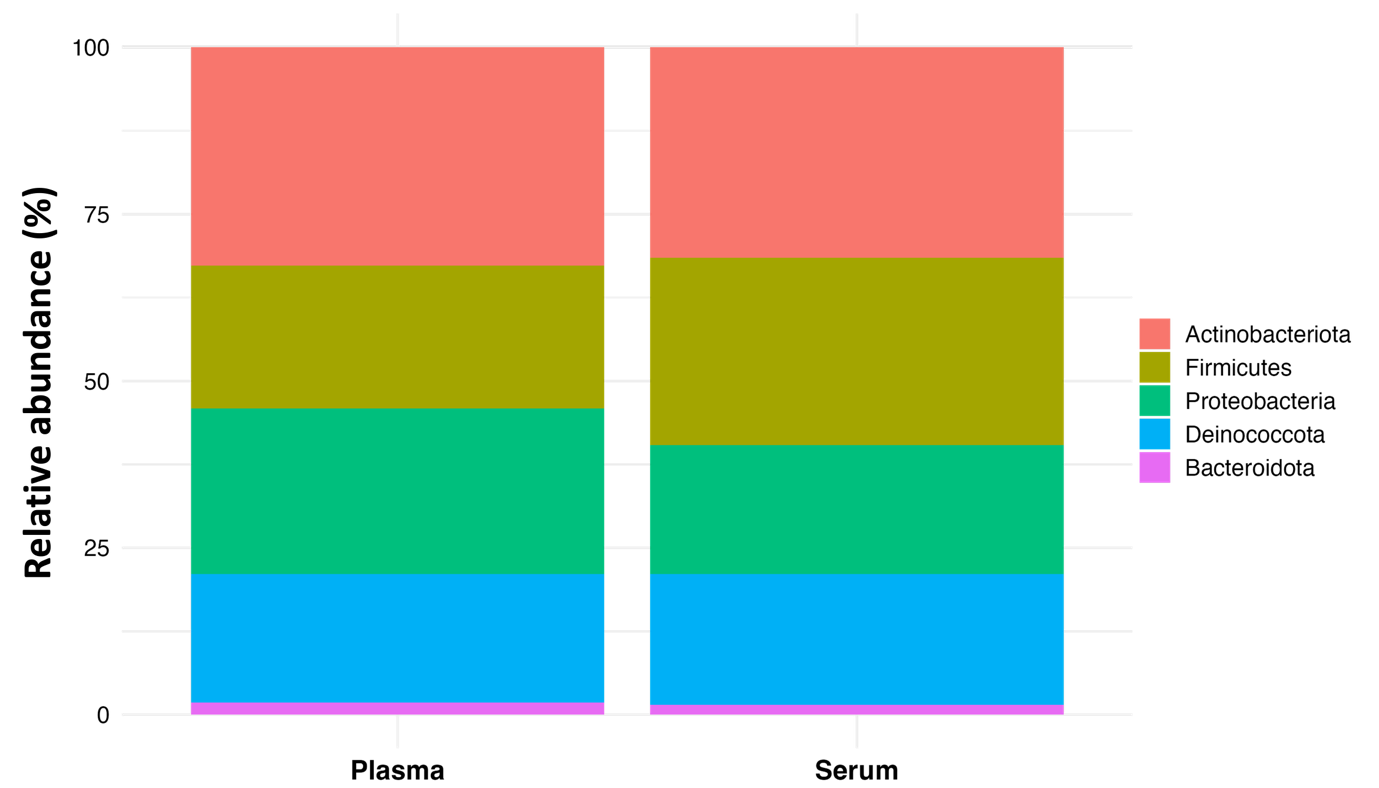


**Supplementary Figure S2. Relative abundance of the major bacterial phyla in plasma and serum; the Test cohort.** The dominant phyla of the circulating microbial DNA; group-level mean relative abundances.


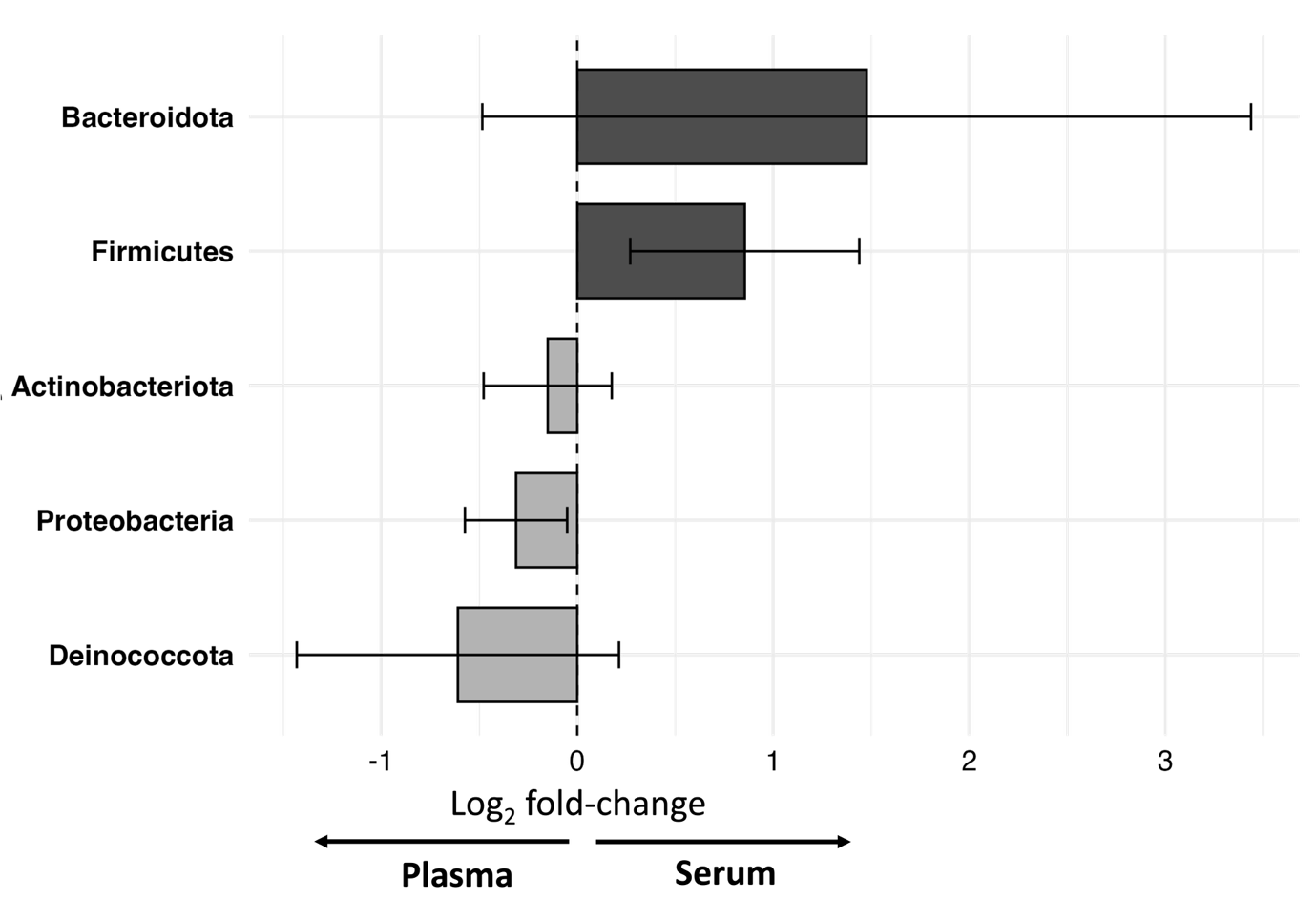


**Supplementary Figure S3. Compositional differences of bacterial phyla between plasma and serum; the Test cohort.** The differential abundance analysis was done in DESeq2 (Love MI *et al., Genome Biol* 2014;15,550). Error bars denote standard error of the log₂ fold-change; adjusted *p* > 0.05 for all phyla.

**Supplementary Figure S4. Beta diversity of the circulating microbial DNA in plasma and serum; the Test cohort.** Principal Coordinates Analysis (PCoA) scores plot based on the Bray-Curtis dissimilarities. Each point represents the composition of the bacterial DNA of one patient (Pt), with the plasma and serum scores connected by a line. The numbers in brackets are the PCoA scores of the total variation.

**
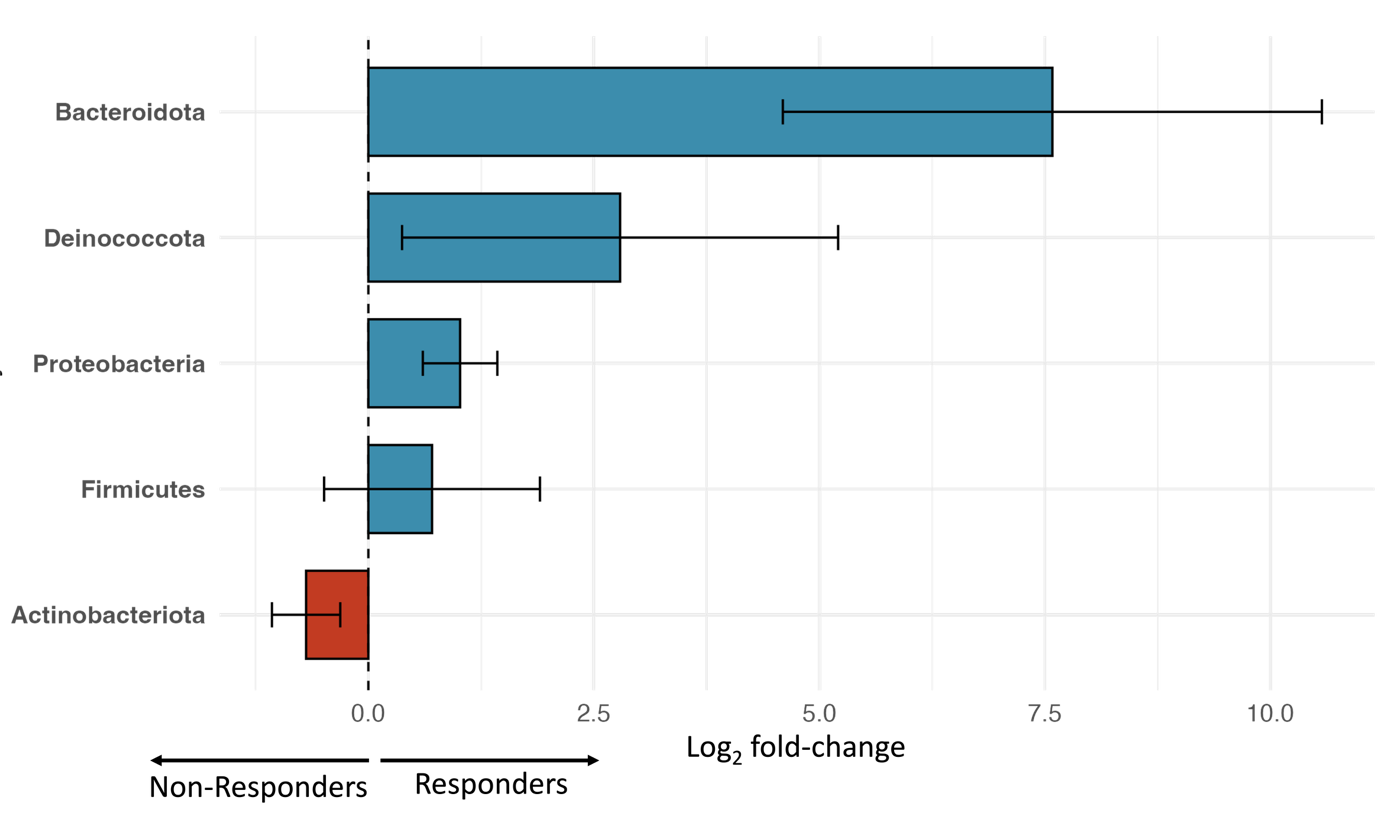
**

**Supplementary Figure S5.** **Compositional differences of bacterial phyla between Responders and Non-Responders to experimental chemoimmunotherapy; the METIMMOX cohort.** Responders: median progression-free survival 32.9 months; Non-Responders: median progression-free survival 2.1 months. The differential abundance analysis was done in DESeq2 (Love MI *et al., Genome Biol* 2014;15,550). Error bars denote standard error of the log₂ fold-change; adjusted *p*-values: *p* = 0.034 for both Bacteriodota and Proteobacteria, *p* > 0.1 for the remaining phyla.

# Supplementary Tables

| **Age (years)** | **Sex** | **BMI (kg/m^2^)** | **Primary tumor site** | **Primary tumor at diagnosis of metastatic disease** | **Time to progression (months)** | **Category** |
| --- | --- | --- | --- | --- | --- | --- |
| 60 | Female | 21.9 | Right colon | Resected | 41.6 | Responder |
| 58 | Male | 27.4 | Right colon | Resected | Not reached at 40.0 | Responder |
| 68 | Female | 24.1 | Right colon | Resected | Not reached at 36.4 | Responder |
| 75 | Female | 27.0 | Right colon | Present | 35.0 | Responder |
| 72 | Female | 36.6 | Right colon | Present | Not reached at 33.1 | Responder |
| 71 | Female | 21.0 | Right colon | Present | 20.7 | Responder |
| 55 | Female | 19.3 | Left colon | Present | 2.6 | Non-Responder |
| 56 | Male | 34.0 | Rectum | Resected | 2.2 | Non-Responder |
| 65 | Female | 27.5 | Left colon | Present | 2.1 | Non-Responder |
| 58 | Male | 32.3 | Right colon | Present | 1.9 | Non-Responder |
| 57 | Female | 21.2 | Right colon | Resected | 1.9 | Non-Responder |

**Supplementary Table S1. Patient and disease characterstics; the METIMMOX cohort.** BMI: body mass index. Patients were given experimental chemoimmunotherapy and categorized as Responders or Non-Responders.

| **Organism** | **Theoretical composition** | **Observed composition; the Test cohort analysis** | **Observed composition; the METIMMOX cohort analysis** |
| --- | --- | --- | --- |
| Listeria monocytogenes | 14.1 | 12.7 | 13.6 |
| Pseudomonas aeruginosa | 4.2 | 7.0 | 7.5 |
| Bacillus subtilis | 17.4 | 16.8 | 17.4 |
| Escherichia coli | 10.1 | 5.5 | 2.9 |
| Salmonella enterica | 10.4 | not detected | not detected |
| Lactobacillus fermentum | 18.4 | 18.3 | 18.5 |
| Enterococcus faecalis | 9.9 | 10.1 | 10.9 |
| Staphylococcus aureus | 15.5 | 15.6 | 15.3 |

**Supplementary Table S2. Theoretical and observed taxonomic composition (in %) of the microbial community DNA standard (ZymoBIOMICS™).**

| **Taxonomic classification** | **The Test cohort analysis** | | **The METIMMOX cohort analysis** | |
| --- | --- | --- | --- | --- |
|  | **Number of reads** | **%** | **Number of reads** | **%** |
| Pseudomonas | 767 | 49.4 | 2365 | 35.8 |
| Cutibacterium | 423 | 27.2 | 2084 | 31.5 |
| Corynebacterium | 148 | 9.5 | 434 | 6.6 |
| Escherichia-Shigella | 75 | 4.8 | 123 | 1.9 |
| Staphylococcus | 67 | 4.3 | 213 | 3.2 |
| Curvibacter | 21 | 1.4 | 80 | 1.2 |
| Unclassified Actinobacteria | 20 | 1.3 | 59 | 0.9 |
| Enhydrobacter | 13 | 0.8 | 98 | 1.5 |
| Unclassified Oxalobacteraceae | 9 | 0.6 | 62 | 0.9 |
| Sphingomonas | 5 | 0.3 | 69 | 1.0 |
| Eukaryota |  |  | 430 | 6.5 |
| Deinococcus |  |  | 59 | 0.9 |
| Anaerococcus |  |  | 188 | 2.8 |
| Psychrobacter |  |  | 98 | 1.5 |
| Chloroplast |  |  | 62 | 0.9 |
| Uncultured Neisseriaceae |  |  | 69 | 1.0 |
| Lactococcus |  |  | 2 | 0.03 |
| Unclassified Dermacoccaceae |  |  | 107 | 1.6 |
| Mycobacterium |  |  | 93 | 1.4 |
| Burkholderia-Caballeronia-Paraburkholderia |  |  | 78 | 1.2 |
| Planococcus |  |  | 51 | 0.8 |
| Nocardioides |  |  | 10 | 0.2 |

**Supplementary Table S3. Taxonomic composition (in %) of the negative (water) controls.**

| **Comparison** | **Phylum** | **Log₂ fold-change** | **Adjusted *p*-value** |
| --- | --- | --- | --- |
| Responders *versus* Non-Responders; all samples | Actinobacteriota | –0.69 | 0.11 |
|  | Bacteroidota | **7.58** | **0.034** |
|  | Deinococcota | 2.79 | 0.31 |
|  | Firmicutes | 0.71 | 0.56 |
|  | Proteobacteria | **1.02** | **0.034** |
| Responders *versus* Non-Responders; Pre samples | Actinobacteriota | –0.69 | 0.36 |
|  | Deinococcota | 4.63 | 0.22 |
|  | Firmicutes | –0.11 | 0.95 |
|  | Proteobacteria | 1.11 | 0.22 |
| Responders *versus* Non-Responders; Post samples | Actinobacteriota | –0.62 | 0.27 |
|  | Deinococcota | 1.53 | 0.60 |
|  | Firmicutes | 1.50 | 0.49 |
|  | Proteobacteria | 0.91 | 0.25 |
| Responders;  Pre *versus* Post samples | Actinobacteriota | –0.46 | 0.93 |
|  | Deinococcota | –0.70 | 0.93 |
|  | Firmicutes | 0.98 | 0.93 |
|  | Proteobacteria | –0.06 | 0.93 |
| Non-Responders;  Pre *versus* Post samples | Actinobacteriota | –0.52 | 0.77 |
|  | Deinococcota | 2.39 | 0.77 |
|  | Firmicutes | –0.64 | 0.77 |
|  | Proteobacteria | 0.13 | 0.77 |

**Supplementary Table S4. Differential abundance analysis of the major plasma bacterial phyla; the METIMMOX cohort.** Plasma samples were collected before treatment initiation (Pre) and after the initial ~2 months of experimental chemoimmunotherapy (Post). Patients were defined as Responders (median progression-free survival 32.9 months) and Non-Responders (median progression-free survival 2.1 months). The differential abundance analysis was done in DESeq2 (Love MI *et al., Genome Biol* 2014;15,550).
